# Effectiveness of Antiviral Therapy in Highly-Transmissible Variants of SARS-CoV-2: A Modeling and Simulation Study

**DOI:** 10.3389/fphar.2022.816429

**Published:** 2022-02-09

**Authors:** Verena Schöning, Charlotte Kern, Carlos Chaccour, Felix Hammann

**Affiliations:** ^1^ Clinical Pharmacology and Toxicology, Department of General Internal Medicine, Inselspital, Bern University Hospital, University of Bern, Bern, Switzerland; ^2^ Graduate School for Health Sciences, University of Bern, Bern, Switzerland; ^3^ Department of Microbiology and Infectious Diseases, Clinica Universidad de Navarra, Pamplona, Spain; ^4^ Centro de Investigaciön Biomédica en Red de Enfermedades Infecciosas, Madrid, Spain; ^5^ ISGlobal, Hospital Clinic,University of Barcelona, Barcelona, Spain

**Keywords:** COVID–19, SARS–CoV-2 variants, variants of concern (VOCs), mathematical disease modeling, molnupiravir, viral kinetic modelling, paxlovid, pharmacometrics

## Abstract

As of October 2021, neither established agents (e.g., hydroxychloroquine) nor experimental drugs have lived up to their initial promise as antiviral treatment against SARS-CoV-2 infection. While vaccines are being globally deployed, variants of concern (VOCs) are emerging with the potential for vaccine escape. VOCs are characterized by a higher within-host transmissibility, and this may alter their susceptibility to antiviral treatment. Here we describe a model to understand the effect of changes in within-host reproduction number R_0_, as proxy for transmissibility, of VOCs on the effectiveness of antiviral therapy with molnupiravir through modeling and simulation. Molnupiravir (EIDD-2801 or MK 4482) is an orally bioavailable antiviral drug inhibiting viral replication through lethal mutagenesis, ultimately leading to viral extinction. We simulated 800 mg molnupiravir treatment every 12 h for 5 days, with treatment initiated at different time points before and after infection. Modeled viral mutations range from 1.25 to 2-fold greater transmissibility than wild type, but also include putative co-adapted variants with lower transmissibility (0.75-fold). Antiviral efficacy was correlated with R_0_, making highly transmissible VOCs more sensitive to antiviral therapy. Total viral load was reduced by up to 70% in highly transmissible variants compared to 30% in wild type if treatment was started in the first 1–3 days post inoculation. Less transmissible variants appear less susceptible. Our findings suggest there may be a role for pre- or post-exposure prophylactic antiviral treatment in areas with presence of highly transmissible SARS-CoV-2 variants. Furthermore, clinical trials with borderline efficacious results should consider identifying VOCs and examine their impact in post-hoc analysis.

## Introduction

With now almost 2 years into the COVID-19 pandemic caused by SARS-CoV-2 there have been abundant drug repurposing efforts. Unfortunately, neither established agents (e.g., hydroxychloroquine) nor experimental drugs have lived up to their initial promise. In fact, only corticosteroids appear to have limited benefits, and then only on the all-cause mortality and need for mechanical ventilation outcomes in severe cases (Siemieniuk et al., 2020). As vaccines are being rolled out worldwide, new virus variants continue to take hold, some of which harbor the potential for vaccine escape (diminished effectiveness of vaccines developed against wild type strains) (Wang et al., 2021). There are indications that immunity acquired during the natural course of a SARS-CoV-2 infection is similarly reduced (Andreano et al., 2021).

Most notorious amongst these variants of concern (VOCs) are five lineages, now termed Alpha through Delta and Omicron (lineages B.1.1.7, B.1.351, P.1, B.1.617.2, and B.1.1.529, respectively). While they were first detected in late 2020 through November 2021, they are assumed to have been circulating for much longer (Rambaut et al., 2020; Tegally et al., 2020; Faria et al., 2021). A hallmark feature of those VOCs is their high transmissibility, in part caused by mutations such as N501Y in the spike protein, resulting in greater affinity for the ACE2 receptor (Baric, 2020).

Public health measures like physical distancing, wearing of personal protective equipment, and stay-at-home orders implemented during outbreaks of wild type strains have contributed to reducing overall transmission, but failed, at least partially, to contain the spread of variants as effectively. First reports of waning vaccine immunity (Goldberg et al., 2021) and mutations compromising vaccine efficacy are appearing, causing disruption to national vaccination strategies, and treatment with convalescent plasma may even select for mutations and new variants (Cohen, 2021; Planas et al., 2021). It is apparent that other preventive measures such as drug-based primary or post-exposure prophylaxis still need to be explored, particularly if viral loads are already high in pre-symptomatic individuals, leading to greater infectiousness (Jones et al., 2021).

The treatment options for COVID-19 appear limited so far, but several antiviral drugs are under consideration. Gilead’s remdesivir, an adenosine nucleotide analog acting as a RNA chain terminator, hence inhibiting viral replication, has been granted emergency use authorization by the FDA for the treatment of COVID-19 patients, with a daily intravenous administration (FDA, 2020). However, the ISARIC WHO study of United Kingdom hospitalized patients with COVID-19, where adults with severe COVID-19 were treated with remdesivir, showed that treatment was not associated with a reduction in mortality (Arch et al., 2021).

Of the many novel antivirals being proposed, two are currently receiving widespread attention. The first, Paxlovid, is a combination of the Pfizer compound PF-07321332 and ritonavir as a pharmacokinetic booster, and acts as an inhibitor of SARS-CoV-2-3-CL protease, thereby reducing viral replication. It has no marketing authorization yet, but preliminary results are promising and show reduction of risk for COVID-19 related hospital admission or death in phase II-III EPIC-HR clinical trials (Mahase, 2021b).

The second, molnupiravir (EIDD-2801 or MK-4482), developed by Merck and Ridgeback, is a nucleoside analog much like remdesivir, but brings with it the advantage of being available as an oral formulation. Molnupiravir is the prodrug (5′-isobutyric ester form) of the nucleoside analog β-D-N^4^-hydroxycytidine (NHC or EIDD-1931) (Painter et al., 2019). Molnupiravir is cleaved in the plasma to liberate NHC. NHC is then phosphorylated in the cell by host kinases to its active form NHC triphosphate (EIDD-2061). The viral RNA-dependent RNA polymerase (RdRp) then inserts the non-functional NHC triphosphate instead of uridine triphosphate. When the resulting RNA is used as template, NHC can form stable base pairs with either guanidine or adenine, leading to what has been termed ‘lethal mutagenesis’ (Kabinger et al., 2021; Malone and Campbell, 2021). *In vitro,* NHC showed an inhibitory effect on SARS-CoV-2 replication in Calu-3 cells, a disease-relevant human lung epithelial cell line, and *in vivo* in a Syrian hamster model (Rosenke et al., 2021). An *in vivo* study in a ferret model showed that, depending on timing of treatment initiation of molnupiravir, the viral replication of SARS-CoV-2 could be completely silences within hours to days. Furthermore, the transmission to uninfected contact animals could be prevented (Cox et al., 2021). These results were corroborated by a study in human lung-only mice, where molnupiravir also markedly inhibited SARS-CoV-2 replication (Wahl et al., 2021). News releases from Merck suggest that molnupiravir is safe in human subjects and that as per interim analysis reduced the risk of hospitalization or death by approximately 50% in patients at risk (Mahase, 2021a).

With this study, we aim to better understand the influence of adaptations in SARS-CoV-2 pathobiology on drug development and treatment. For this, we simulated the impact of changes of within-host reproduction number R_0_, as proxy for within-host transmissibility, on antiviral treatment efficacy. For this purpose, we used a recent model of within-host viral kinetics trained on load profiles from Singapore obtained in early 2020 (Kern et al., 2021), and combined it with a pharmacometric model to simulate treatment with molnupiravir as a representative of novel experimental antivirals currently under investigation for the treatment of COVID-19. We then modified R_0_ in the viral kinetics model and assessed the effects on within-host viral dynamics. The assessed antiviral effect metrics are total viral load (the total amount of virions produced calculated as area under the viral load curve (AUC)), peak viral load (the highest amount of virions as maximum Ct_min_), and disease duration (time above the detection threshold, virus permanence time).

## Methods

### Data Sources

Young et al. (2020), a study that followed 18 COVID-19 patients in four Singapore hospitals, provided the viral kinetic patient profiles. Authors used RT-PCR to determine the viral load from nasopharyngeal swabs and results were provided as cycle threshold (Ct) values. Because the correlation between Ct values and viral load changes depending on the laboratory and analytics, model output was related with observed Ct values with a published regression fit (Chu et al., 2020). An average incubation period of 5 days was chosen for all patients, because the time of infection was not recorded (Lauer et al., 2020). Positivity threshold was set at 35, corresponding to 101.58 copies/mL (Wang et al., 2020).

### Viral Kinetics Models

Viral loads were simulated from a target-cell limited model, a dynamic in-host infection model considering target cells, infected cells and virus particles (Ciupe and Heffernan, 2017). The equilibrium states, stability and characterization of different initial conditions are demonstrated in other studies (Abuin et al., 2020; 2021). We added the dynamics of an acquired immune response around 10 days post inoculation (dpi). In brief, a small amount of virus particles *V* are considered to infect a pool of target cells *T* with cellular infection rate *β*. Infected cells *I* produce and shed virions at a production rate *p* (Canini and Perelson, 2014). The rate parameters *c* and *δ* represent viral clearance, and cell death of infected cells, respectively. The time-dependent number of target cells (Eq. 1), infected cells (Eq. 2) and circulating virions (Eq. 3) are described by the following system of ordinary differential equations:

$$\frac{dT}{dt}=\;-\beta TV$$ (1)

$$\frac{dI}{dt}=\beta TV-\;\delta I\;$$ (2)

$$\frac{dV}{dt}=\left(1-\eta \right)pI-c\left(1+\varepsilon_{immunity}\right)V$$ (3)

Acquired immunity ε_immunity_ develops according to a sigmoidal E_max_ model (Eq. 4), and effects of drug treatment η enter dependent on their concentrations and IC_50_ or EC_50_ values for their respective targets (Eq. 5). We modeled drug treatment within an impulsive antiviral framework where *C(t),* the concentration of the drug and its effects at a given time dynamically changes in accordance with the pharmacokinetics of molnupiravir (Hernandez-Mejia et al., 2020; Perez et al., 2021):

$$\varepsilon_{immunity}=\frac{E_{max\;\;immunity}\;\times \;t^{Hill_{immunity}}}{EC_{50\;immunity}^{Hill_{immunity}}+\;t^{Hill_{immunity\;}}\;}$$ (4)

$$\eta =\;\frac{E_{max}\;\times C\left(t\right)}{IC_{50}+C\left(t\right)}$$ (5)

The within-host reproduction number R_0_ is the amount of cells infected per infected cell. It is not a single variable in the system of ordinary differential equations used to describe the viral kinetic, but depends on the other variables. We used a modern approach for invasion analysis (Hurford et al., 2010) for calculating R_0_ (Eq. 6), which was already applied by another study to model Sars-CoV-2 viral kinetics (Czuppon et al., 2020):

$$R_{0}=\frac{p\beta T_{0}}{\delta \left(c+\beta T_{0}\right)}$$ (6)

Table 1 shows viral kinetics model parameters used. A detailed description of the model and its implementation is given in Kern et al. (2021).

**TABLE 1:** Viral kinetics and molnupiravir pharmacokinetic model parameters. The pharmacokinetic profile of NHC was extracted from a safety study in fasted healthy volunteers given a single dose of 800 mg molnupiravir (Painter et al., 2021) with a digitizing software. A one compartment model with transit absorption and linear elimination was fitted to the pharmacokinetic profile. *IC_50_ value for NHC (EIDD-1931) inhibition of SARS-CoV-2 replication in human lung epithelial Calu-3 cells after 24 h treatment.

Parameter	Definition	Value	Reference
Within-host viral kinetics			
*β*	Cellular infection rate	$\beta =\frac{R_{0}c\delta }{T_{0}\left(p-R_{0}\delta \right)}$	Calculated
*δ*	Infected cell death rate	0.54 day^−1^	Estimated
*p*	Viral production rate	10.2 (copies/mL) day^−1^ cell^−1^	Estimated
*c*	Viral clearance	5.07 day^−1^	Estimated
*R*_*0*_	Within-host reproduction number	3.79	Li et al. (2020)
*T*_*0*_	Initial target cells	10^5^ cells	Fixed by authors
*V*_*0*_	Initial virus load (inoculum)	10^0^ virions	Fixed by authors
*EC*_*50, immunity*_	Half maximal effective concentration for immune response	10.2	Estimated Long et al. (2020)
*Hill*_*immunity*_	Slope of dose-response curve	3.4	Estimated Long et al. (2020)
*E*_*max*_ *,*_*immunity*_	Maximum effect on viral clearance	57.0	Estimated
Pharmacokinetics of molnupiravir			
Ktr	Transit absorption rate	2.01 h^−1^	Estimated
Mtt	Mean transit time	0.64 h	Estimated
ka	Absorption rate constant	2.01 h^−1^	Estimated
V	Central compartment volume	0.052 L	Estimated
Cl	Clearance	0.11 L/h	Estimated
*IC_50_	Half maximal inhibitory concentration	0.4146 µM	Rosenke et al. (2021)
PPB	Plasma protein binding	13.3%	Estimated van Prroijen et al. (1977)

### Highly Transmissible SARS-CoV-2 Variants

Highly transmissible variants were considered to have 1.25-, 1.5-, and 2-fold increases in the within-host reproductive number R_0_ compared to the wild type (R_0_ = 3.79). Co-adaptation (i.e., a less transmissible mutation) was accounted for by simulating a 0.75-fold decrease in R_0_.

### Pharmacokinetic Models

No published population pharmacokinetics model was available for molnupiravir (EIDD-2801/MK-4482), the prodrug of NHC (EIDD-1931). The pharmacokinetic profile of NHC was extracted from a safety study in fasted healthy volunteers given single doses of 200–800 mg molnupiravir (Painter et al., 2021) with a digitizing software. We used Monolix (version 2020R1), to fit the following model to the pharmacokinetic profile: a one compartment model with linear elimination, an extravascular administration with transit compartments and first order absorption. Table 1 shows the pharmacokinetic model parameters. We performed a non-compartmental analysis (NCA) with the resulting curve to estimate common pharmacokinetic metrics (see Supplementary Table S2).

Only the unbound fraction of the drug should be considered available for target engagement. To date, no plasma protein binding values are available for molnupiravir or NHC. Since NHC has a structure close to cytidine and cytidine in turn has a similar structure to cytosine arabinoside (Ara-C), we used findings from a study on the interaction of Ara-C with human plasma proteins, where 13.3% of Ara-C in the plasma was bound to proteins (van Prroijen et al., 1977). The effective drug concentrations were reduced accordingly for simulations of drug effects.

In addition to protein binding, organ distribution also needs to be considered. We used literature-based approximations to adjust for differences between plasma and lung concentration profiles. Murine pharmacokinetics of NHC and the active form NHC triphosphate (EIDD-2061) after an oral dose of 500 mg/kg NHC has been described by Painter et al. (2019). Concentration curves were available in mice spleen, brain, heart, kidney, liver and lung for NHC and the activated metabolite. After extracting the curves for each organ, PK parameters (AUC, C_max_, T_max_) were computed. For the lung, C_max_ for NHC (EIDD-1931) was 47.99 nmol/g and for NHC triphosphate (EIDD-2061) was 7.87 nmol/g. The metabolite/parent drug ratio (NHC triphosphate/NHC) was 0.164, and was used to estimate the available concentrations of active metabolite over time.

### Pharmacodynamic Effects

The effectiveness of NHC was shown *in vitro* in Calu-3 cells (human lung epithelial cell line) by Rosenke et al. (2021), after 24 h of treatment with NHC. An IC_50_ of 0.4146 µM was used in the simulations as an inhibitory influence on viral production *p*, as molnupiravir inhibits viral replication. The proposed dosing regimen was molnupiravir 800 mg every 12 h for 5 days (Merck Sharp & Dohme Corp, 2020).

### Software

Pharmacokinetics data for NHC and NHC triphosphate was read out with WebPlotDigitizer (version 4.2, https://automeris.io/WebPlotDigitizer). Corresponding pharmacokinetic profiles were modelled in Monolix (version 2020R1, http://www.lixoft.com, Antony, France) and simulated in GNU R (version 3.6.3, R Foundation for Statistical Computing, http://www.R-project.org, Vienna, Austria), with the corresponding R package mlxR (version 4.1.3). PK parameters for the mice organs were calculated in the Non Compartmental Analysis framework of PKanalix (version 2020R1, http://www.lixoft.com, Antony, France). Data analysis and visualization were performed with GNU R. Ordinary differential equation (ODE) systems were implemented with the R package deSolve (version 1.28).

## Results

The viral load profiles of 13 untreated patients [from (Young et al., 2020)] were best described by a standard target cell limited model. Death rate of infected cells *δ*, viral production rate *p,* viral clearance *c*, and maximum immune effect on clearance E_max, immunity_ were estimated from the individual profiles with the Nelder-Mead method and then averaged (Kern et al., 2021). The pharmacokinetics of molnupiravir based on digitalized plasma concentration curves, estimated from clinical trial data analysis, have a satisfactory fit with the information provided in Painter et al. (2021) (see Supplementary Figure S1). Table 1 contains the final viral kinetics and molnupiravir pharmacokinetics model parameter estimates.

**FIGURE 1:**
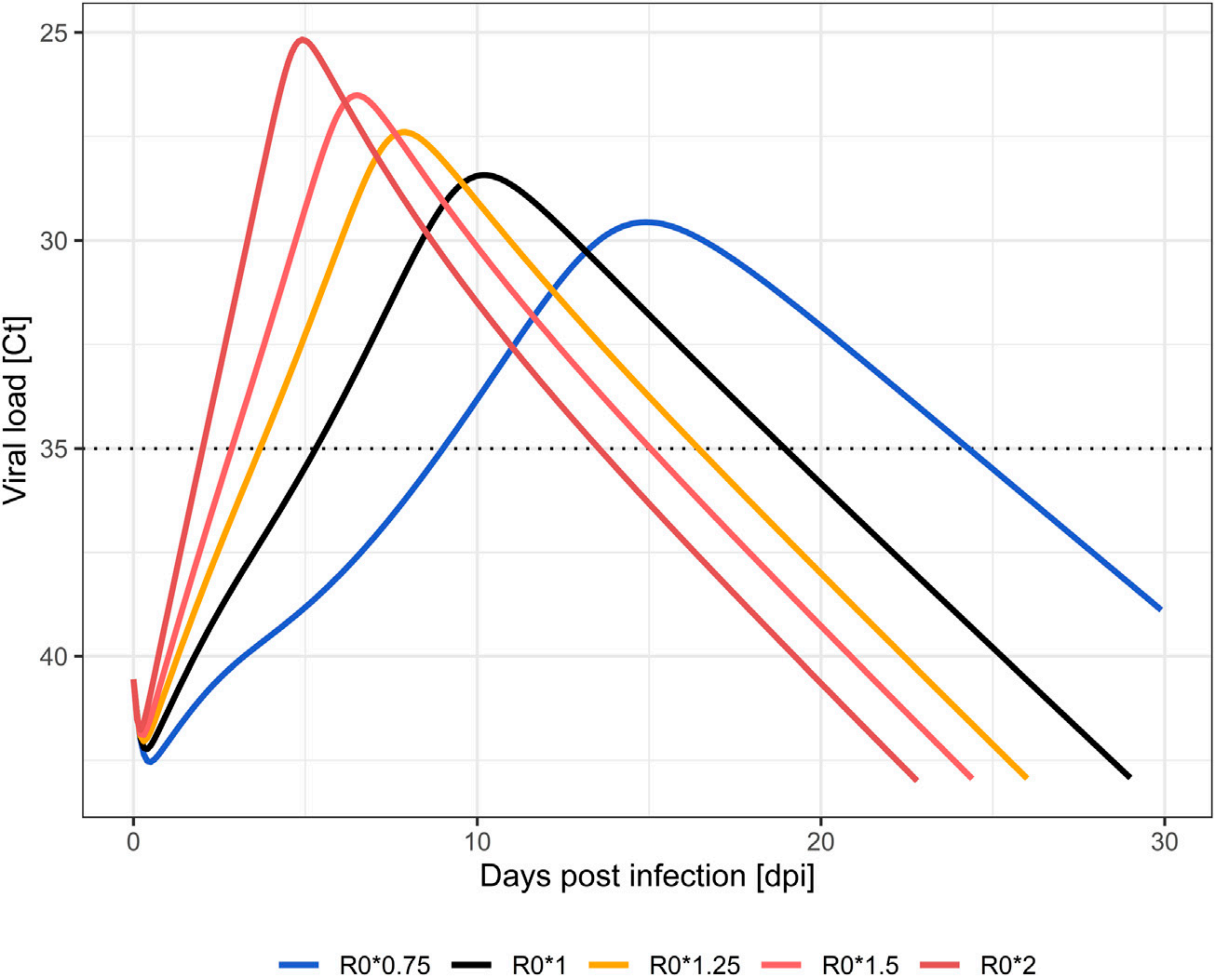
Simulated viral load profiles by change in within-host infectivity (R_0_). Wild type SARS-CoV-2 strain (black), less transmissible (blue), highly transmissible (orange to red) variants. Limit of quantification (Ct = 35) is displayed as a dotted line.

Viral load dynamics using wild type (R_0_*1) parameterizations served as the baseline for comparison. Viral loads reach positivity (Ct < 35) after 5.4 days. Positivity is maintained for a total duration of 13.5 days, and the viral load peak of 28.4 Ct_min_ appears after 8.1 days (Kern et al., 2021). Compared to wild type strains, increases in within-host R_0_ up to two-fold transmissibility resulted in earlier positivity (2.1–3.7 dpi *vs.* 5.4 dpi in wild type). Higher viral load peaks (Ct_min_ 25.2–27.4 *vs*. 28.4 in wild type) are achieved earlier after inoculation (4.5–6.3 vs 8.1 days in wild type) and, while reduced, durations below the Ct threshold of 35 are comparable (11.4–12.7 *vs.* 13.5 days in wild type). Total viral load [area under the viral load curve (AUC)] increased 152–402% in comparison to wild type. Co-adaptation to 75% the original R_0_ was predicted to result in a later positivity at 9.1 dpi with a prolonged duration of 15.1 days, a lower viral load peak of Ct_min_ 29.6 at 11.9 days, and a reduced viral load AUC (66%). Simulated profiles are shown in Figure 1; Table 2.

**TABLE 2:** Simulated within-host viral kinetics at different levels of cellular infectivity (given as multiples of R_0_ = 3.79). Abbreviations: days post infection (dpi); day (d); minimum serial cycle threshold values (Ct_min_); time of Ct_min_ (T_max_); area under the curve (AUC); percentage difference in AUC compared to wild-type (ΔAUC%).

Infectivity	Start positivity [dpi]	Duration [d]	Ct_min_	T_max_	AUC [d*log (copies/mL)]	ΔAUC%
Wild type						
R_0_	5.4	13.5	28.4	8.1	12003	100
Highly transmissible mutation						
R_0_*1.25	3.7	12.7	27.4	6.3	18278	152
R_0_*1.5	2.9	12.1	26.5	5.4	26648	222
R_0_*2	2.1	11.4	25.2	4.5	48197	402
Less transmissible mutation						
R_0_*0.75	9.1	15.1	29.6	11.9	7975	66

The treatment effects of molnupiravir are modeled within an impulsive antiviral framework. Molnupiravir 800 mg is administered every 12 h for 5 days, however, the concentration needed for antiviral activity within in the model cannot be sustained over the time between administrations based on the EC_50_ applied and the pharmacokinetic parameters. Therefore, effects of drug treatments are visible in the viral load curve by repeated sharp decreases and increases of viral load (see Supplementary Figure S2).

**FIGURE 2:**
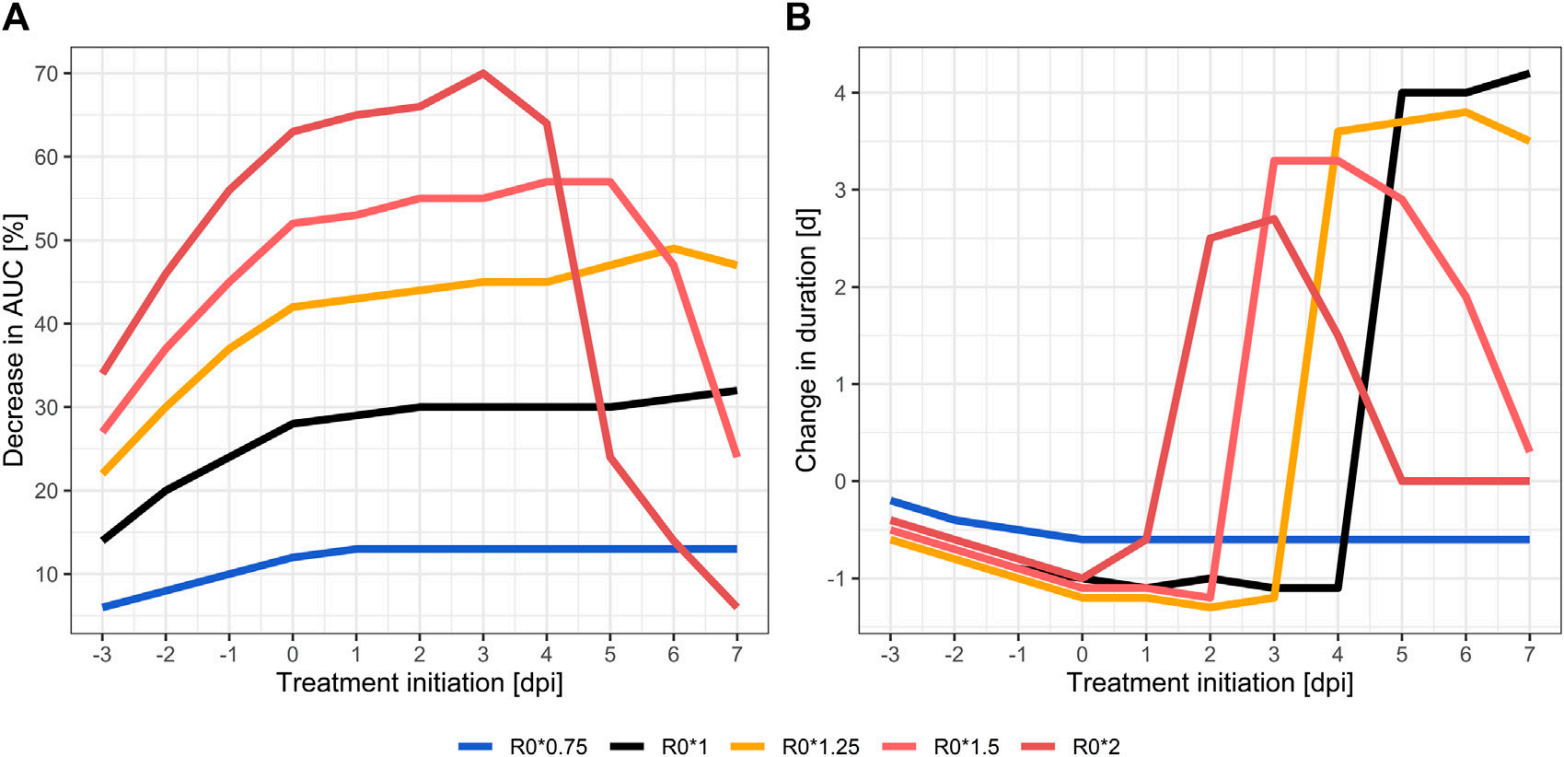
Relative treatment effect in different VOCs compared to natural (untreated) course: relative changes of total viral load as area under the viral load curve (AUC) **(A)** and relative virus permanence time (time above serological positivity threshold) **(B)**. Wild type SARS-CoV-2 strain (black), less transmissible (blue), highly transmissible (orange to red) variants.

Treatment with molnupiravir 800 mg every 12 h for 5 days in wild type (R_0_*1) leads to a reduced total viral load and peak viral load down to 68% and 0.8 log units, respectively. Treatment initiation before five dpi resulted in a delayed positivity (i.e., meeting the detectability criterion of Ct < 35). In addition, the total duration was shorter if treatment was initiated before five dpi, and longer if initiated later. These treatment effects were sensitive to changes in R_0_ (Figure 2). Higher transmissibility was positively correlated with a greater reduction in total viral load, peak viral load and delayed time to positivity when treated early. Compared to untreated courses, the total viral load is reduced to 51% (R_0_*1.25) or 30% (R_0_*2) in the highly-transmissible variants. Ct_min_ levels were reduced by more than two log units in R_0_*2 (27.7 *vs*. 25.2 untreated) and viral peak loads were reached at a later time point. Duration was less sensitive to changes in R_0_, with a tendency towards prolonged positivity with increasing R_0_ (R_0_*1.25: 16.5 *vs.* 12.7 days untreated, R_0_*2: 14.1 *vs*. 11.4 days untreated). Furthermore, the treatment effects were not only influenced by transmissibility, but also by timing of treatment initiation, i.e. a higher the transmissibility requires an earlier treatment initiation. Later treatment initiation diminishes the antiviral effect of the treatment quickly. In less transmissible variants, the positive treatment effects were less pronounced. The total viral load and peak viral load were only reduced by up to 13% and 0.1 log units, respectively. On the other hand, the time windows for maximal treatment effect were wider, and later treatment initiation therefore did not sacrifice much efficacy (Supplementary Table S1).

## Discussion

With this modeling and simulation analysis of SARS-CoV-2 variants, we show the potential influence of altered within-host transmissibility on patient viral load dynamics. While the study is *in silico* and not based on *in vivo* data, the results suggest that patients infected with a VOC are likely to experience shorter, but stronger exposures to virions with higher peak loads. This is confirmed by a recent publication, which showed that patients infected with the Delta variant had approximately 1′000-fold higher viral loads than what was seen in earlier 19A/19B strains (Li et al., 2021). Similar evidence exists also for other VOCs (Frampton et al., 2021).

Early treatment of SARS-CoV-2 infections achieved the greatest effect on total viral load and peak load, independently from the antiviral treatment studied, as previously noted (Goncalves et al., 2020; Kim et al., 2020; Kern et al., 2021). As time of treatment initiation nears the peak of viral loads when untreated, when most of the susceptible target cells are already infected, antiviral effect is increasingly attenuated. This can be seen in the steep decline of impact on total viral load in Figure 2. The more target cells are already infected due to a delayed treatment initiation, the less intracellular viral replications can be prevented by the antiviral treatment. This effect is accentuated in highly transmissible VOCs, which appear to be even more susceptible to an early initiation of antiviral treatment. The higher transmissibility of VOC leads to a quicker infection of all target cells, which can be seen as steep ascent in Figure 1. Consequentially, positivity in RT-PCR testing in highly transmissible VOC is expected to be achieved about 3 days sooner than in wild type, also the peak viral concentrations is reached earlier (see Supplementary Table S1). Due to the high viral replication dynamic, especially in the early days of infection, the simulation predicts that a timely initiated antiviral treatment is more effective than in variants with low transmissibility, i.e., a lower viral replication dynamic. Support for this is seen in the manufacturer’s early termination of the inpatient part of placebo-controlled phase II/III dose-finding trials (MOVe-IN in inpatients, and MOVe-OUT in outpatients) out of lack of efficacy (Merck, 2021a). MOVe-out, on the other hand, which was halted after an interim analysis of 775 patients, found a reduction of risk for hospitalization or death from 14.1 to 7.3% in at-risk, non-hospitalized adult patients with mild-to-moderate COVID-19 (number needed to treat = 15) (Merck, 2021b).

Containing the COVID-19 pandemic requires multiple strategies, including vaccination, prophylaxis and treatment, combined with diagnostic, quarantine and contact tracing, in addition to protective measures like social distancing, mask wearing and hand hygiene. Any delay in making a decision-to-treat because of lack of symptoms or availability of diagnostic reports may result in worse outcomes. Possible scenarios include pre-exposure prophylaxis in isolated outbreaks or post-exposure prophylaxis for at-risk contacts of SARS-CoV-2 positive patients, e.g., specific settings such as residential homes, in immunosuppressed people, or localized outbreaks. However, rapid diagnostics combined with contact-tracing strategies may provide a more realistic alternative to treatment of outpatients before the onset of clinical symptoms.

Furthermore, the impulsive effects of the antiviral treatment in our model oscillates between complete suppression of viral replication to no antiviral effect. Provided that an actual population pharmacokinetics had been available, it be possible to optimize the treatment schedule, as shown for the treatment of influenza A with zanamivir (Hernandez-Mejia et al., 2020).

Several limitations need to be considered. Measurements of viral dynamics neither directly translate to clinical courses nor to individual infectiousness, though such correlations have been described (Fajnzylber et al., 2020; He et al., 2020). We simulated from a model that accounts for acquired immune response as described in mid 2020 in patients from hospitals in Chongqing (near Hubei Province). Immunogenicity of VOCs may differ, as may the response of other populations. Additionally, predicting the effects of prophylaxis is difficult with the type of model employed here as extinction (complete disappearance of virions from the system) is a fringe case. We modelled the population pharmacokinetics of molnupiravir based on digitalized plasma concentration curves, as no other information was publicly available. Even though pharmacokinetic parameters estimated from actual trial data analysis are bound to be more precise, the resulting curves fit well with the information provided in (Painter et al., 2021). As we used molnupiravir as exemplary drug to analyse the interaction of VOCs and antiviral treatment, slight changes in the pharmacokinetic parameters might result in different AUC, peak load and duration values, but do not change the overall conclusion of the study.

Published versions of molnupiravir clinical trial protocols do not provide information on pharmacokinetic distribution parameters such as protein binding or transporter interactions. We assumed protein binding to only marginally limit the availability of free drug (similar to structure analogues or nucleoside reverse transcriptase inhibitors used for other indications such as HIV/AIDS) (Ridgeback Biotherapeutics, 2020). Cellular uptake of nucleosides is transporter-dependent (Pastor-Anglada and Pérez-Torras, 2018), and this might also limit the concentration of the active substance of molnupiravir within the respiratory tissue: in a mouse model, the concentration of NHC-triphosphate plateaued at 10 nmol/g lung tissue, even at higher plasma concentrations (Yoon et al., 2018). No comparable data are available in humans, so it remains unclear whether a saturation effect is observable in human lung tissue at the recommended clinical doses. As a consequence, should a relevant plateau effect exist, the model may overestimate molnupiravir efficacy. However, this limitation only applies to our model, as clinical data have shown the efficacy of molnupiravir (Mahase, 2021a), suggesting sufficient tissue concentrations.

The authors would like to stress that molnupiravir was used as a model drug to study the effects on altered within-host infectivity in SARS-CoV-2, not as an endorsement for its use or proof of its clinical efficacy as a prophylactic drug or treatment in manifest cases. We ran comparable simulations with Paxlovid, which also inhibits viral replication, and obtained comparable results (data not shown) with regards to within-host viral dynamics and antiviral drug efficacy. Even though we were not able to model remdesivir, another inhibitor of viral replication, we assume that changes in transmissibility may lead to similar changes in within-host viral dynamics and antiviral drug efficacy.

Apart from focusing efforts on early treatment, running clinical trials should account for variants in trial design and analysis. Given that many trials have included subjects during a time when the now discovered VOCs had already been circulating, it could be worthwhile to revisit borderline efficacious drugs and screen patient samples for these mutant strains in order to perform subgroup analyses, focusing on responders vs. non-responders. This may uncover significant effects against certain variants even when no effect was seen on trial level. Additionally, clinical trials should consider a subgroup analysis based on days after symptom onset before treatment initiation, as this influences the efficacy of the treatment. This would also improve the comparability of different antiviral clinical trials, if the inclusion criteria vary in this point. If successful, this would open up additional avenues for early treatment or prophylaxis that could complement vaccine campaigns in areas of high VOC prevalence, especially as long as these are still suffering from production shortages and supply chain problems. Furthermore, the problem of vaccine resistance and escape should be considered because it can negatively affect public health, as vaccination campaigns could retroactively be rendered ineffective. With case incidences on the rise again even in populations with high vaccine coverage, prophylactic treatment could help prevent hospital overcrowding.

## Data Availability

The original contributions presented in the study are included in the article/Supplementary Material, further inquiries can be directed to the corresponding author. The source code is available on GitHub: https://github.com/cptbern/sars2-variants.
