# Supplementary material for: Effectiveness of Antiviral Therapy in Highly-Transmissible Variants of SARS-CoV-2: A Modeling and Simulation Study
Source: Front Pharmacol. 2022 Feb 9;13:816429. doi: 10.3389/fphar.2022.816429 (PMC8864116; doi:10.3389/fphar.2022.816429)
Supplement: Supplementary file 1 [file DataSheet1.docx]

Supplementary Material

**Supplementary Table S1.** Simulated effects of treatment with 800 mg molnupiravir treatment every 12h for 5 days by infectivity (given as multiples of R_0_ = 3.79) and time of treatment initiation. Abbreviations: days post infection (dpi); day (d); minimum serial cycle threshold values (Ct_min_); time of Ct_min_ (T_max_); area under the curve (AUC); percentage difference in AUC compared to wild-type (ΔAUC%).

| **Infectivity** | **Treatment initiation [dpi]** | **Start positivity [dpi]** | **Duration [d]** | **Ct_min_** | **T_max_** | **AUC [d*log(copies/mL)]** | **ΔAUC%** |
| --- | --- | --- | --- | --- | --- | --- | --- |
| **wild type** |  |  |  |  |  |  |  |
| **R_0_** | -3 | 7.0 | 13.0 | 28.7 | 9.7 | 10321 | 86 |
|  | -2 | 7.9 | 12.8 | 28.8 | 10.6 | 9649 | 80 |
|  | -1 | 8.8 | 12.6 | 28.9 | 11.5 | 9094 | 76 |
|  | 0 | 9.7 | 12.5 | 29.0 | 12.4 | 8644 | 72 |
|  | 1 | 10.0 | 12.4 | 29.0 | 12.7 | 8513 | 71 |
|  | 2 | 10.1 | 12.5 | 29.0 | 12.9 | 8444 | 70 |
|  | 3 | 10.3 | 12.4 | 29.0 | 13.1 | 8390 | 70 |
|  | 4 | 10.4 | 12.4 | 29.0 | 13.2 | 8361 | 70 |
|  | 5.4 | 5.4 | 17.5 | 29.0 | 13.1 | 8342 | 70 |
|  | 6 | 5.4 | 17.5 | 29.1 | 11.2 | 8321 | 69 |
|  | 7 | 5.4 | 17.7 | 29.2 | 9.0 | 8173 | 68 |
|  | untreated | 5.4 | 13.5 | 28.4 | 8.1 | 12003 | 100 |
| **highly transmissible mutants** | |  |  |  |  |  |  |
| **R_0_*1.25** | -3 | 5.3 | 12.1 | 27.8 | 7.9 | 14333 | 78 |
|  | -2 | 6.1 | 11.9 | 28.0 | 8.7 | 12820 | 70 |
|  | -1 | 6.9 | 11.7 | 28.2 | 9.6 | 11595 | 63 |
|  | 0 | 7.8 | 11.5 | 28.3 | 10.5 | 10620 | 58 |
|  | 1 | 8.1 | 11.5 | 28.4 | 10.8 | 10335 | 57 |
|  | 2 | 8.3 | 11.4 | 28.4 | 11.0 | 10170 | 56 |
|  | 3 | 8.4 | 11.5 | 28.4 | 11.1 | 10044 | 55 |
|  | 4 | 3.7 | 16.3 | 28.5 | 9.4 | 9963 | 55 |
|  | 5.4 | 3.7 | 16.4 | 28.7 | 7.2 | 9651 | 53 |
|  | 6 | 3.7 | 16.5 | 29.2 | 6.9 | 9305 | 51 |
|  | 7 | 3.7 | 16.2 | 28.1 | 6.4 | 9721 | 53 |
|  | untreated | 3.7 | 12.7 | 27.4 | 6.3 | 18278 | 100 |
| **R_0_*1.5** | -3 | 4.3 | 11.6 | 27.0 | 6.9 | 19405 | 73 |
|  | -2 | 5.1 | 11.4 | 27.3 | 7.7 | 16723 | 63 |
|  | -1 | 5.9 | 11.2 | 27.5 | 8.5 | 14583 | 55 |
|  | 0 | 6.7 | 11.0 | 27.8 | 9.3 | 12910 | 48 |
|  | 1 | 7.0 | 11.0 | 27.8 | 9.6 | 12422 | 47 |
|  | 2 | 7.2 | 10.9 | 27.9 | 9.8 | 12119 | 45 |
|  | 3 | 2.9 | 15.4 | 27.9 | 8.5 | 11869 | 45 |
|  | 4 | 2.9 | 15.4 | 28.1 | 6.8 | 11491 | 43 |
|  | 5.4 | 2.9 | 15.0 | 28.1 | 5.7 | 11554 | 43 |
|  | 6 | 2.9 | 14.0 | 26.9 | 5.4 | 14166 | 53 |
|  | 7 | 2.9 | 12.4 | 26.5 | 5.4 | 20222 | 76 |
|  | untreated | 2.9 | 12.1 | 26.5 | 5.4 | 26648 | 100 |
| **R_0_*2** | -3 | 3.4 | 11.0 | 25.9 | 5.8 | 31689 | 66 |
|  | -2 | 4.1 | 10.8 | 26.2 | 6.6 | 25892 | 54 |
|  | -1 | 4.8 | 10.6 | 26.5 | 7.3 | 21383 | 44 |
|  | 0 | 5.6 | 10.4 | 26.9 | 8.1 | 17955 | 37 |
|  | 1 | 5.5 | 10.8 | 27.0 | 8.3 | 16975 | 35 |
|  | 2 | 2.5 | 13.9 | 27.1 | 7.3 | 16181 | 34 |
|  | 3 | 2.1 | 14.1 | 27.7 | 5.4 | 14435 | 30 |
|  | 4 | 2.1 | 12.9 | 26.7 | 4.6 | 17245 | 36 |
|  | 5.4 | 2.1 | 11.4 | 25.2 | 4.5 | 36696 | 76 |
|  | 6 | 2.1 | 11.4 | 25.2 | 4.5 | 41609 | 86 |
|  | 7 | 2.1 | 11.4 | 25.2 | 4.5 | 45434 | 94 |
|  | untreated | 2.1 | 11.4 | 25.2 | 4.5 | 48197 | 100 |
| **less transmissible mutation** | | | | | | | |
| **R_0_*0.75** | -3 | 10.7 | 14.9 | 29.6 | 13.5 | 7513 | 94 |
|  | -2 | 11.7 | 14.7 | 29.7 | 14.5 | 7319 | 92 |
|  | -1 | 12.6 | 14.6 | 29.7 | 15.4 | 7151 | 90 |
|  | 0 | 13.6 | 14.5 | 29.7 | 16.4 | 7006 | 88 |
|  | 1 | 13.9 | 14.5 | 29.8 | 16.7 | 6963 | 87 |
|  | 2 | 14.0 | 14.5 | 29.8 | 16.8 | 6943 | 87 |
|  | 3 | 14.2 | 14.5 | 29.8 | 17.0 | 6927 | 87 |
|  | 4 | 14.2 | 14.5 | 29.8 | 17.0 | 6918 | 87 |
|  | 5.4 | 14.3 | 14.5 | 29.8 | 17.1 | 6914 | 87 |
|  | 6 | 14.3 | 14.5 | 29.8 | 17.1 | 6913 | 87 |
|  | 7 | 14.3 | 14.5 | 29.8 | 17.1 | 6911 | 87 |
|  | untreated | 9.1 | 15.1 | 29.6 | 11.9 | 7969 | 100 |

**Supplementary Table S2.** Estimated pharmacokinetic parameters of a single dose of 800 mg molnupiravir.

| **Parameter** | **Description** | **Estimated value** |
| --- | --- | --- |
| T_max_ | Time of peak concentration | 1.03 h |
| C_max_ | Peak concentration | 3413 ng/mL |
| Half-life | The time required for the concentration of the drug to reach half of its original value | 1.39 h |
| AUC | Area under the curve from the time of dosing to the last measurable concentration | 9043 ng/mL.h^-1^ |
| CL/F | Clearance (CL) over bioavailability (F) based on concentration at the final observation time | 0.087 L.h^-1^ |
| V/F | Volume of distribution (V) over bioavailability (F) | 0.18 L |


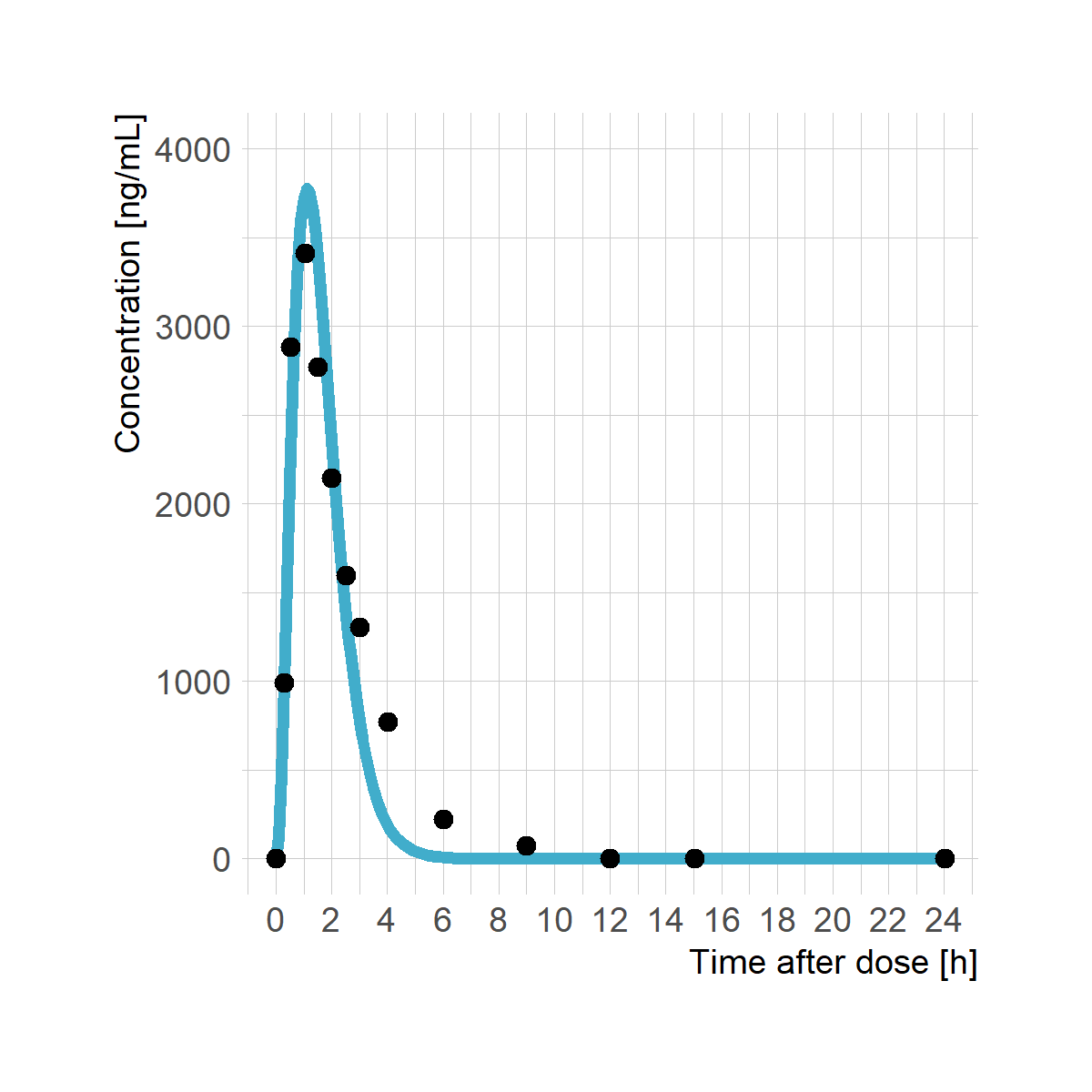


**Supplementary Figure S1**: Measured plasma concentrations and fitted curve for NHC (EIDD-1931). Comparison of the fitted pharmacokinetics curve (blue line) with the original pharmacokinetics observations (black points) of single dose 800 mg molnupiravir (Painter et al., 2021) with PKanalix version 2020R1.

| 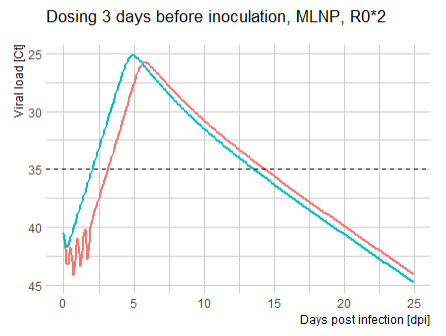 | 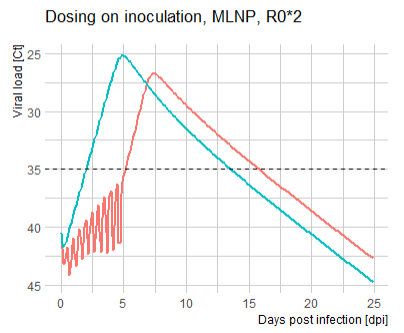 | 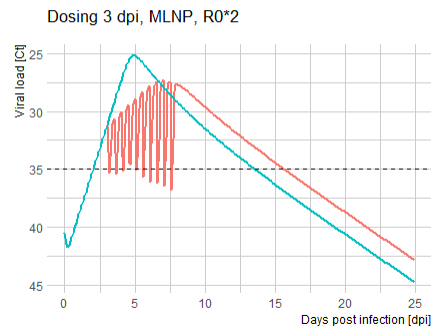 |
| --- | --- | --- |
|  | 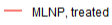 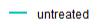 |  |

**Supplementary Figure S2:** Treatment related impulsive changes of viral load dynamics. Impulsive changes of viral load dynamics of SARS-CoV-2 variant (R0*2) due to 10x800mg molnupiravir treatment (red line) compared to untreated (blue line) for different treatment initiation.

# References

Painter, W.P., Holman, W., Bush Jim, A., Almazedi, F., Malik, H., Eraut Nicola, C.J.E., Morin Merribeth, J., Szewczyk Laura, J., and Painter, G.R. (2021). Human Safety, Tolerability, and Pharmacokinetics of Molnupiravir, a Novel Broad-Spectrum Oral Antiviral Agent with Activity against SARS-CoV-2. *Antimicrobial Agents and Chemotherapy* 65**,** e02428-02420.
